# Concomitant Intra-Aortic Balloon Pump Throughout Extracorporeal Cardiopulmonary Resuscitation: A Meta-Analysis of Cohorts

**DOI:** 10.31083/RCM45096

**Published:** 2026-03-10

**Authors:** Huiruo Liu, Liangshan Wang, Hongfu Fu, Hong Wang, Xing Hao, Zhongtao Du, Chenglong Li, Xiaotong Hou

**Affiliations:** ^1^Centre for Cardiac Intensive Care, Beijing Institute of Heart, Lung and Blood Vessel Diseases, Beijing Anzhen Hospital, Capital Medical University, 100029 Beijing, China

**Keywords:** extracorporeal cardiopulmonary resuscitation, cardiac arrest, intra-aortic balloon pump, meta-analysis

## Abstract

**Background:**

Inconsistent reports exist regarding the efficacy of using a concomitant intra-aortic balloon pump (IABP) among cardiac arrest (CA) patients undergoing extracorporeal cardiopulmonary resuscitation (ECPR). Thus, this review was conducted to summarize the prognoses of adult ECPR patients with concurrent IABP.

**Methods:**

Data were gathered from PubMed, Embase, MEDLINE, Web of Science, and Cochrane databases. Cohorts of adult patients receiving ECPR with or without IABP, reporting short-term mortality, neurological outcomes, or extracorporeal membrane oxygenation (ECMO) weaning rates, were recruited. Characteristics of the study population and the above-mentioned outcomes were extracted. A random-effects model was used to pool the data. Subgroup analyses were conducted in the propensity score-matching (PSM) population.

**Results:**

Nine cohorts with 5260 adult ECPR patients were included. In-hospital/30-day mortality, neurological performances of survivors, and ECMO weaning outcomes were not significantly different between populations with and without IABP. Nevertheless, younger patients with IABP showed an apparent improvement in in-hospital/30-day mortality. Similar findings were demonstrated in the analyses of PSM cohorts. High heterogeneity was present in the total cohort.

**Conclusions:**

In ECPR populations, concomitant IABP did not influence short-term survival, neurological, or ECMO weaning outcomes in the total cohort. However, IABP exhibited a survival benefit in the younger ECPR population. Further research in specific populations is warranted to validate and endorse our aggregated data.

**The PROSPERO Registration:**

CRD42024528761, Registration Link: https://www.crd.york.ac.uk/PROSPERO/view/CRD42024528761.

## 1. Introduction

Extracorporeal cardiopulmonary resuscitation (ECPR) refers to the application of venoarterial extracorporeal membrane oxygenation (VA-ECMO) alongside conventional cardiopulmonary resuscitation for refractory cardiac arrest, which has been associated with improved prognosis [1, 2]. However, despite the immediate provision of sufficient circulatory support, evidence indicates that VA-ECMO can contribute to elevated wall tension and oxygen consumption, as well as blood stasis and ventricular arrhythmias, hindering cardiac recovery [3, 4, 5].

Intra-aortic balloon pump (IABP), a feasible and valid percutaneous strategy for left ventricular (LV) unloading, may provide more physiologically pulsatile blood flow to vital organs during VA-ECMO support [6, 7]. An up-to-date meta-analysis of cohorts in the domain of cardiac shock showed improved prognosis with the addition of IABP to VA-ECMO [8]. In addition, Impella, another efficient LV unloading approach, is associated with improved survival and neurological outcomes in pooled analyses [9].

The current view on additional IABP support in ECPR remains controversial, owing to inconsistent conclusions from multiple existing cohorts and a lack of relevant meta-analysis. Thus, we conducted a pooled analysis to describe the treatment efficacy of additional IABP during ECPR in adult populations with cardiac arrest.

## 2. Methods

This meta-analysis was reported in accordance with the Preferred Reporting Items for Systematic reviews and Meta-Analyses (PRISMA) guidelines [10]. The study protocol has been recorded in the PROSPERO registry (ID: CRD42024528761, Registration Link: https://www.crd.york.ac.uk/PROSPERO/view/CRD42024528761).

### 2.1 Literature Search

A search was performed in PubMed, Embase, MEDLINE, Web of Science, and Cochrane databases until March 23rd, 2024, by three independent investigators, with no language restriction, utilizing the combination of the following strings: “heart arrest”, “extracorporeal membrane oxygenation”, and “intra-aortic balloon pump”. Manual hand-searching of reference lists from pertinent reviews was supplemented, and certain trials regarding cardiogenic shock (CS) were screened because data on patients with cardiac arrest might be reported as subgroup analyses. The full search strategy was expanded in **Supplementary Table 1**.

### 2.2 Selection Criteria

As per the Population, Exposure, Comparator, and Outcomes (PECO) principle, the inclusion criteria were set as follows: (P) participants: adult individuals who administrated ECPR support after cardiac arrest (CA); (E) exposures and (C) comparators: with or without IABP performed after ECMO pump-on; (O) outcomes: in-hospital/30-day mortality, favorable neurological performance in survivors, or ECMO weaning. The neurologically favorable outcome was defined as achieving a Cerebral Performance Category (CPC) scale score of 1 or 2, with CPC encompassing the following five gradings: (1) favorable cerebral recovery, (2) moderate cerebral impairment, (3) severe cerebral disability, (4) coma or vegetative state, and (5) death or brain death. Exclusion criteria included: (1) studies that are not longitudinal cohorts; (2) studies published in languages other than English. If there was overlap in study populations from the same registry or group, only the largest sample was included.

### 2.3 Data Extraction and Quality Assessment

Two independent investigators extracted and cross-checked data from the retrieved studies, resolving any discrepancies by discussion or referral to a third investigator. The extracted data were as follows: (1) name of first author, research region, number of centers, study design, and duration; (2) characteristics of patients, including sample size, age, sex, CA-associated data, medical history, and in-hospital medications; (3) ratios of propensity score-matched (PSM), and outcomes reported. The quality of the studies was assessed using the Newcastle–Ottawa scale (NOS) [11].

### 2.4 Statistics Analysis

Odds ratios (ORs) and 95% confidence intervals (CIs) were used as the primary indicators of association between IABP support and outcomes among populations undergoing ECPR. Before the pooled analyses, the ORs were log-transformed, and the standard errors were derived from the 95% CIs. Heterogeneity was assessed using Cochran’s Q test and I^2^ statistics. Statistical models were selected based on heterogeneity *p*-values: a random-effects model was used when 0.05 ≤ *p* < 0.5, and pooled ORs were not reported for *p* < 0.05 due to substantial heterogeneity. Results from PSM cohorts were further pooled. Publication bias was assessed using funnel plots and Egger’s test. Sensitivity analyses stratified by center were conducted for analyses involving more than two studies. RevMan (Version 5.4; The Nordic Cochrane Centre, Copenhagen, Denmark) software was used to conduct these analyses.

## 3. Results

### 3.1 Search Results

**Fig. 1.**
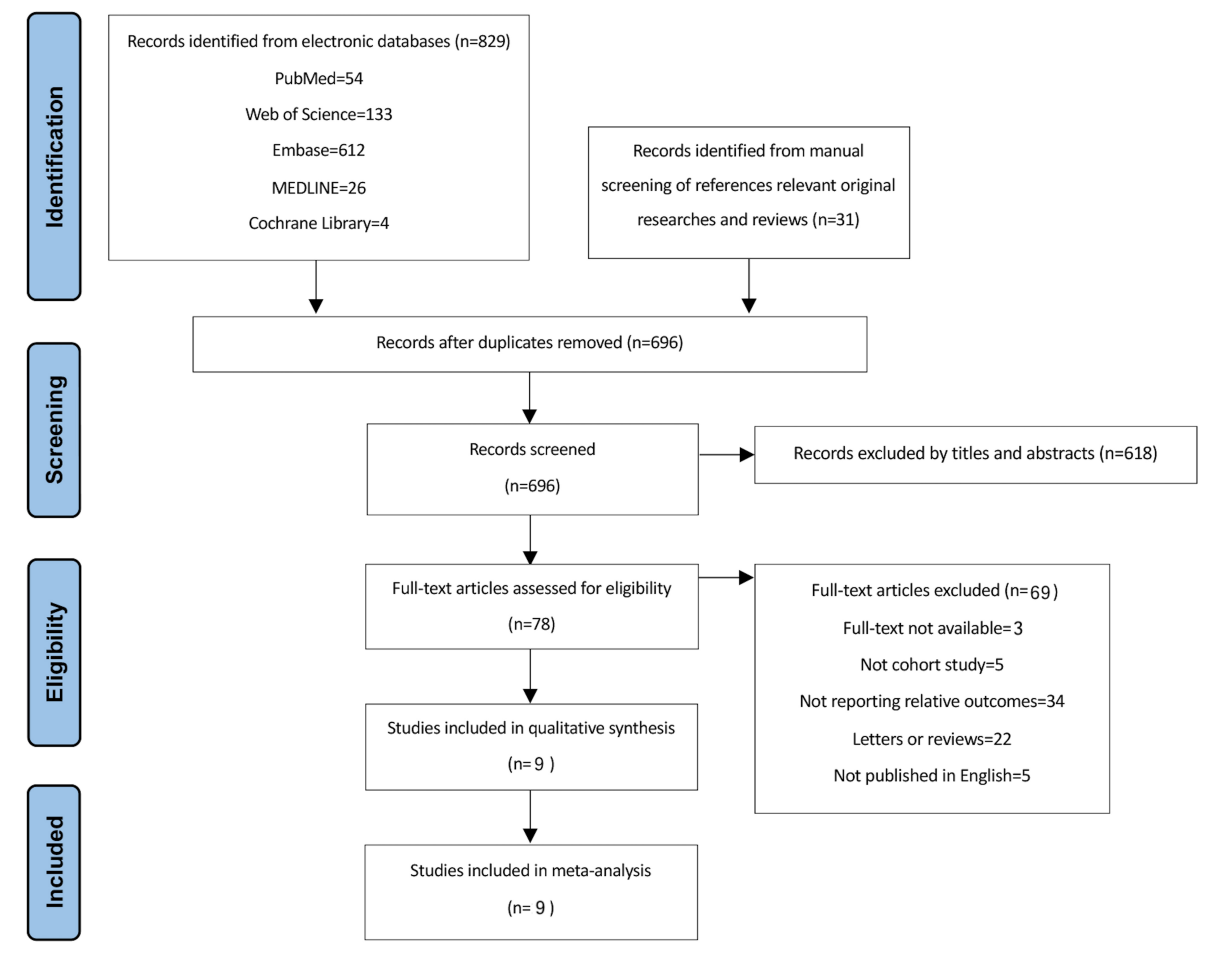
**PRISMA flowchart of search strategy**.

Fig. 1 shows the employed protocol in the literature review. A total of 860 articles were initially searched from databases, and after eliminating duplicates, 78 papers were subjected to full-text examination. Finally, nine cohorts were included for further analysis [12, 13, 14, 15, 16, 17, 18, 19, 20].

The included cohorts were conducted in 264 care centers and published from 2012 to 2024, with a mean study period of 7.3 years; 263 centers (99.6%) were located in Asian countries, and only one center (0.4%) was in the United States. Five studies were multicenter-designed [12, 15, 16, 18, 19]. All cohorts, except for two [15, 19], were retrospectively performed, out of which four reported results from propensity-matched groups (Table 1, Ref. [12, 13, 14, 15, 16, 17, 18, 19, 20]). All studies had NOS scores of 9, indicating high quality (**Supplementary Table 2**).

**Table 1.** **Study characteristics**.

Study	Design	Region	Study duration	Study center(s)	Ratios of PSM	Outcomes
Kagawa *et al*. 2012, [12]	RC	Japan	January 2004 to May 2011	2 (Hiroshima City Asa Hospital, Hiroshima City Hospital)	N/A	Mortality
Aoyama *et al*. 2014, [13]	RC	Japan	August 1993 to August 2000	1 (Kitasato University Hospital)	N/A	Mortality
Guru *et al*. 2015, [14]	RC	USA	May 2001 to December 2014	1 (N/A)	N/A	Mortality
Kim *et al*. 2016, [15]	PC	Korea	November 2005 to April 2014	50 (from Korea Acute Myocardial Infarction Registry)	1:1	Mortality
Kuroki *et al*. 2021, [16]	RC	Japan	January 2010 to December 2017	74 (from Tokyo Cardiovascular Care Unit Network database)	1:1	Mortality, neurological performance
Chen *et al*. 2022, [17]	RC	Korea	January 2004 to December 2013	1 (Samsung Medical Centre)	1:1	Mortality, neurological performance, ECMO weaning
Kashiura *et al*. 2023, [18]	RC	Japan	June 2014 to December 2019	73 (from Japanese Association for Acute Medicine OHCA (JAAM-OHCA) registry)	1:1	Mortality, neurological performance
Xu *et al*. 2023, [20]	RC	China	July 2018 to September 2022	1 (Hunan Provincial People’s Hospital)	N/A	ECMO weaning
Li *et al*. 2024, [19]	PC	China	January 2017 to May 2022	61 (from CSECLS registry)	N/A	Mortality

RC, retrospective cohort; PC, prospective cohort; N/A, not applicable; PSM, propensity score matching; OHCA, out-of-hospital cardiac arrest; ECMO, extracorporeal membrane oxygenation; CSECLS, Chinese Society of Extracorporeal Life Support.

### 3.2 Study Population

Across the nine included studies, sample sizes ranged from 38 to 2135 for patients who underwent ECPR. In all, 5260 adult individuals undergoing ECPR were enrolled, of whom 2684 (51.0%) received IABP after VA-ECMO pump-on, and 2576 (49.0%) did not. Patients receiving IABP tended to be male (81.5% vs. 68.8%; *p* < 0.01), older (64.3 y vs. 63.3 y; *p* < 0.01), and were more likely to experience shockable electrocardiogram rhythms (55.9% vs. 49.6%; *p* < 0.01). Moreover, percutaneous coronary intervention (PCI) was more frequently performed in patients with ECPR (60.5% vs. 16.9%; *p* < 0.01). In contrast, the ECPR only and ECPR + IABP groups showed similar rates of out-of-hospital cardiac arrest (*p* = 0.14) and acute myocardial infarction (AMI) (*p* = 0.99), as well regarding similar proportions of individuals with prior myocardial infarction (MI) (*p* = 0.89), diabetes mellitus (*p* = 0.60), and hypertension (*p* = 0.14) (**Supplementary Table 3**).

### 3.3 Outcomes

**Fig. 2.**
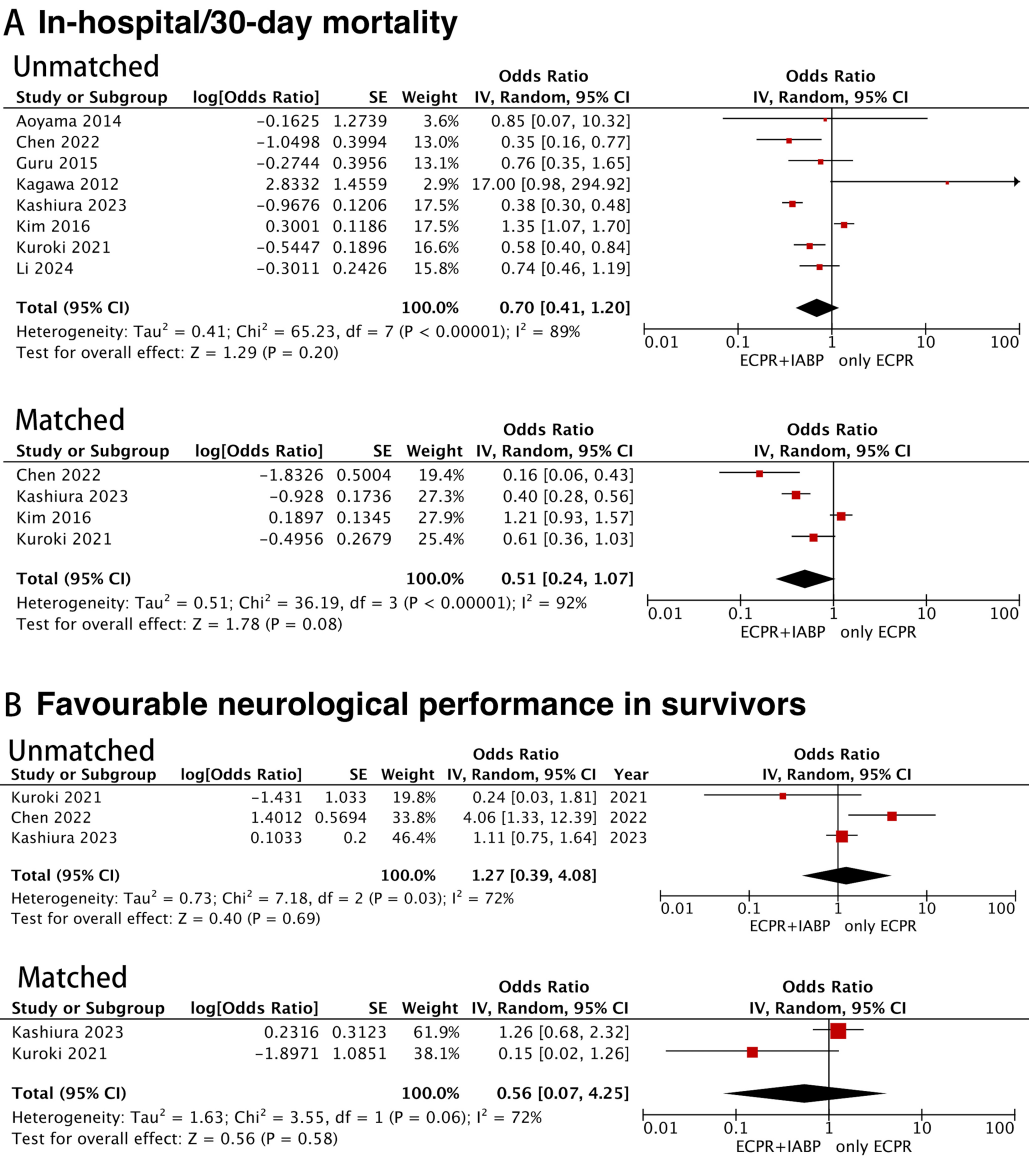
**Forest plots for the meta-analysis in the total cohort and the propensity score-matched cohort**. (A) Meta-analysis of in-hospital/30-day mortality. (B) Meta-analysis of favorable neurological performance in survivors. SE, standard error; IV, inverse variance; CI, confidence interval; ECPR, extracorporeal cardiopulmonary resuscitation; IABP, intra-aortic balloon pump.

**Fig. 3.**
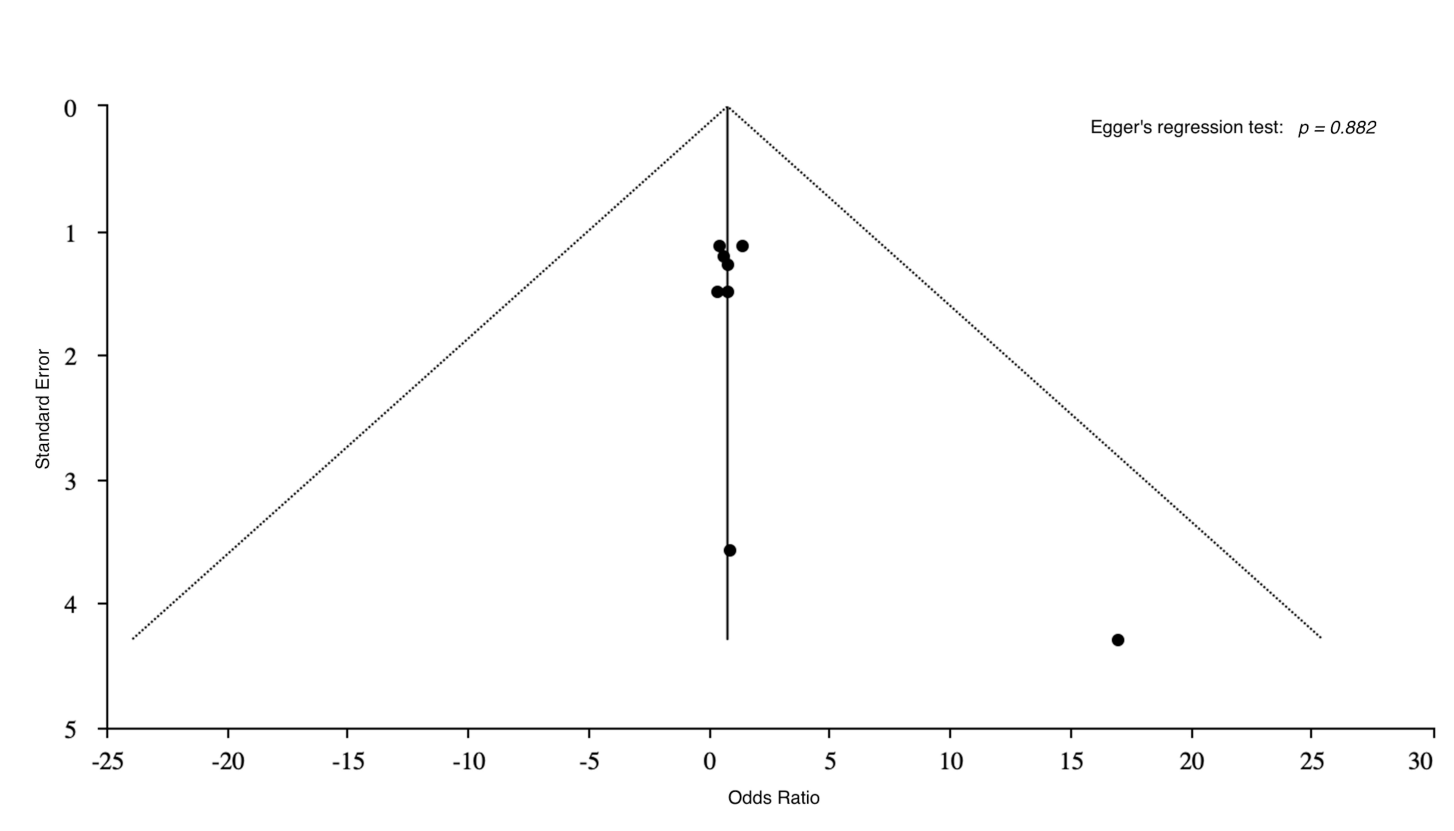
**Funnel plots for the publication bias**.

Overall, the pooled analyses across total cohorts indicated that patients with additional IABP support had in-hospital/30-day mortality and neurological outcomes similar to those receiving ECPR alone. Consistent findings were observed when the records of PSM cohorts were pooled (OR for neurological outcomes: 0.56, 95% CI: 0.07–4.25; *p* = 0.58) (Fig. 2). No apparent asymmetry of the funnel plot was shown (Egger’s regression test: *p* = 0.882) (Fig. 3).

Furthermore, there were no statistically significant differences between these two groups across the rates of successful ECMO weaning (**Supplementary Fig. 1**).

### 3.4 Sensitivity Analyses

Sensitivity analyses indicated that the combination of ECPR and IABP was not stably associated with improved in-hospital/30-day survival, except when the Kim study [15] was excluded. In that case, the ECPR + IABP group demonstrated significantly lower in-hospital/30-day mortality in both the original and PSM cohorts (OR: 0.39, 95% CI: 0.22–0.67; *p* < 0.01). Moreover, the overall findings regarding neurological outcomes remained robust (**Supplementary Fig. 2**).

## 4. Discussion

Across this comprehensive meta-analysis of 5260 patients receiving VA-ECMO during CPR owing to refractory cardiac arrest, approximately 51% of the patients received IABP. Concomitant IABP did not affect short-term mortality, neurological performance, or weaning rate from VA-ECMO in this pooled cohort. Notably, among patients of younger age, IABP combined with ECPR was significantly linked to 5.2% lower mortality in comparison to those only on ECPR.

Despite the theoretical benefits of mechanical circulatory support (MCS) in refractory CA, there remains a scarcity of high-quality evidence to guide the use of MCS [21, 22]. A recent survey of the National Inpatient Sample registry revealed a gradual decrease in IABP utilization in the context of CA; however, IABP remained the preferred device for these populations [21]. This is mainly due to a lack of robust evidence of the practical benefits of IABP. Notably, similar challenges have persisted in the field of CS, where the class of recommendation for IABP has been continuously downgraded, and even recent international guidelines have shifted toward advising against routine IABP in CS populations [23]. Recent large-scale data have shown improved post-arrest survival and neurological outcomes following VA-ECMO support during CA, leading to an increasing tendency to use VA-ECMO in the management of CA [24, 25]. Consistent with these findings, our pooled study cohort demonstrated a trend toward greater VA-ECMO utilization in cases of CA, with five of the nine studies published in the last 5 years. The VA-ECMO had several pathophysiological advantages for treating CA, including rapid bedside access for high-risk coronary interventions, adequate cardiac output support of 3–5 L, and simultaneous support of cardiac and pulmonary function [26], making VA-ECMO a broader application for refractory CA in clinical settings. Nevertheless, in the context of reduced LV contractility during refractory CA, VA-ECMO would contribute to a further elevation of afterload that could result in exacerbation of LV performance, and consequently cause a series of pathological consequences, such as decreased coronary perfusion pressure, pulmonary edema, or even pneumorrhagia, thrombogenesis, blood stasis, ventricular arrhythmias etc., impairing myocardial recovery [27].

Given the clinical reality that restoring adequate perfusion with VA-ECMO comes at the cost of increased LV afterload, strategies for LV unloading are increasingly proposed to prevent or treat complications associated with VA-ECMO [4, 28]. Both IABP and Impella are commonly used MCSs in ECPR, yet no evidence-based guidelines for these devices have been published. Theoretically, IABP and Impella can both partially unload LV workload, reducing myocardial oxygen consumption and improving coronary perfusion during diastole, as demonstrated in preclinical models [7, 29]. However, only reduced mortality and good neurological outcomes in relation to Impella were found by Thevathasan *et al*. [9] from 13 study records at 32 hospitals comprising a total of 1014 ECPR individuals. In contrast, no significant improvements in survival or neurological prognosis associated with concomitant IABP were observed in our current meta-analysis of 5260 ECPR adults from 264 centers. The rationale for the noted differences between the Thevathasan *et al*. reviews [9] and ours is multifold. First, despite the theoretical advantages of IABP in providing counterpulsation support, the effectiveness of IABP is influenced by multiple factors. IABP relies on residual cardiac function and is limited by high extracorporeal blood flow through the VA-ECMO circuit. Additionally, tachycardia, which frequently occurs in the setting of CA, could also diminish the counterpulsation effect by altering the IABP assist ratio. In contrast, Impella can pump blood from the LV to the ascending aorta using a miniature axial flow pump independent of cardiac function, providing greater unloading capacity and flow rates that typically exceed the maximum 0.8–1 L/min achievable with an IABP [30]. Notably, a previous meta-analysis showed that IABP can improve outcomes in patients with AMI-induced CS, likely due to enhanced residual cardiac function compared with those with refractory CA [31]. However, the landmark IABP-SHOCK II trial [32], which demonstrated no survival benefit with IABP use in CS, underscored the potential limitations of IABP in certain clinical scenarios. Second, the age characteristics of the pooled ECMELLA cohort (LV unloading with Impella in addition to VA-ECMO) met the current international ECPR guideline criteria, with an average age of 56.0 years (younger age) [33]; nonetheless, our study population skews relatively older, with an average age of approximately 60 years. Importantly, after excluding the study by Kim *et al*. [15], which focused on patients aged nearly 70 years, the IABP has demonstrated compelling survival benefits. These suggest that the potential advantages of concomitant IABP may also be realized by close adherence to the ECPR guidelines, including consideration of age. Third, compared with Impella, IABP behavior is influenced by pre-IABP pulse pressure, while a previous study found that patients with a pulse pressure exceeding 10 mmHg had superior neurological perfusion [34]. Thus, if further homogeneity studies could be conducted based on the magnitude of the pressure differential, additional advantages of IABP may be uncovered.

Currently, no RCTs describe the effect of IABP in ECPR. Our meta-analysis, which used PSM to adjust for confounders, found no significant benefit with concomitant IABP use. On one hand, given the physiological principles of IABP [35], it may be more beneficial in younger patients or those with an ischemic etiology. Conversely, the considerable heterogeneity among patients with CA, particularly in those who progress to subsequent CS, highlights the complexity of managing this patient population. Specifically, the use of VA-ECMO in CS remains controversial, as evidenced by the recent ECLS-SHOCK trial [36], which failed to demonstrate a survival benefit. Therefore, well-designed trials are urgently required to establish clear indications for IABP use in ECPR and to translate the theoretical advantages of IABP into robust clinical evidence. Crucially, clinicians should consider and balance against the potentially increased risks of complications during dual MCS, for instance, major bleeding, limb ischemia, acute kidney injury, arterial laceration, etc., which could have an apparent hit on survival benefits [37, 38]. Hence, it is essential to monitor ECPR patients undergoing dual MCS closely.

## 5. Limitations

The current study had some limitations. First, apparent heterogeneity among studies was revealed. High-quality trials evaluating IABP during ECPR remain scarce, which may contribute to the lack of robust clinical guidance and, consequently, to variability in decision-making across institutions. Notably, center experience and case volume may critically affect patient outcomes [39]. Another explanation might be the selection of all CA types. In particular, CA complicating AMI and non-ischemic myocardial diseases differ fundamentally in their etiology, treatment, and prognosis [40]. Additionally, most studies do not report real-time ECMO data, such as blood flow, sweep gas flow, and oxygen/air ratios. Furthermore, since these settings are typically determined at the discretion of the treating physician, patient group comparability across studies is further limited. Second, our systematic review only included observational cohorts that are prone to confounding by indications, because patients requiring IABP were always sicker at baseline. Moreover, the majority of individuals were drawn from Asian cohorts, likely attributed to declining IABP use and a corresponding rise in Impella utilization in Europe following the negative outcomes of the IABP-SHOCK II trial [41]. In contrast, in the Asia region, where many countries are developing, Impella adoption remains limited by high costs and challenges in disseminating technology and providing clinical training [42], resulting in the continued predominance of IABP. To minimize bias arising from baseline incomparability, we used a random-effects model to pool data, conducted subgroup analyses within PSM cohorts, and performed sensitivity analyses. Third, due to data deficiency, the sample size was too small, and the detailed baseline information could not be obtained, which hindered further subgroup analyses based on age, sex, causes of disease, etc., to explore the effect of IABP among certain specific populations. Finally, our study primarily focused on short-term prognosis, with limited explorations of long-term survival and functional recovery.

## 6. Conclusions

In the current meta-analysis of 5260 adult patients suffering from refractory CA undergoing ECPR, the concomitant IABP did not affect short-term mortality, neurological outcomes, or weaning rate from VA-ECMO. However, improved survival benefits with IABP were observed in the younger age group. Given the data from our study, further randomized controlled trials (RCTs) are needed among specific populations.

## Availability of Data and Materials

All data could be obtained from this published article and its supplementary materials.

## Supplementary Material

Supplementary material associated with this article can be found, in the online version, at https://doi.org/10.31083/RCM45096.
