# Supplementary material for: Concomitant Intra-Aortic Balloon Pump Throughout Extracorporeal Cardiopulmonary Resuscitation: A Meta-Analysis of Cohorts
Source: Rev Cardiovasc Med. 2026 Mar 10;27(3):45096. doi: 10.31083/RCM45096 (PMC13036546; doi:10.31083/RCM45096)
Supplement: Supplementary file 1 [file 2153-8174-27-3-45096-s1.zip › Supplementary Material.docx]

**Supplementary Materials**

Supplementary Fig. 1 …………………………………………………………………………2

Supplementary Fig. 2 …………………………………………………………………………3

Supplementary Table 1 …………………………………………………………………………4

Supplementary Table 2 …………………………………………………………………………9

Supplementary Table 3 …………………………………………………………………………10

**Supplementary Fig. 1.** ECMO weaning.


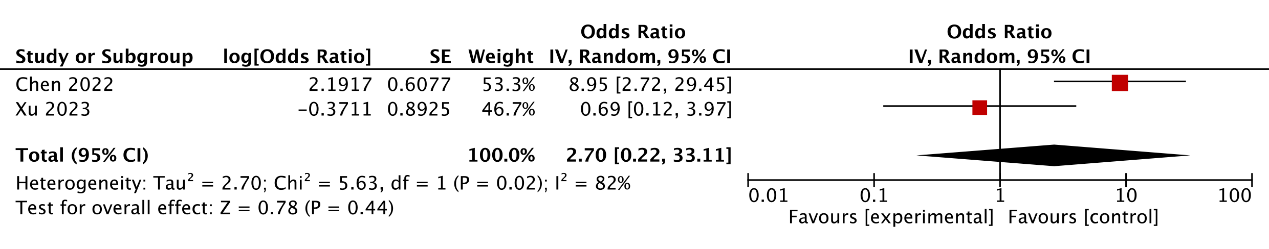


**Supplementary Fig. 2.** Sensitivity analysis.


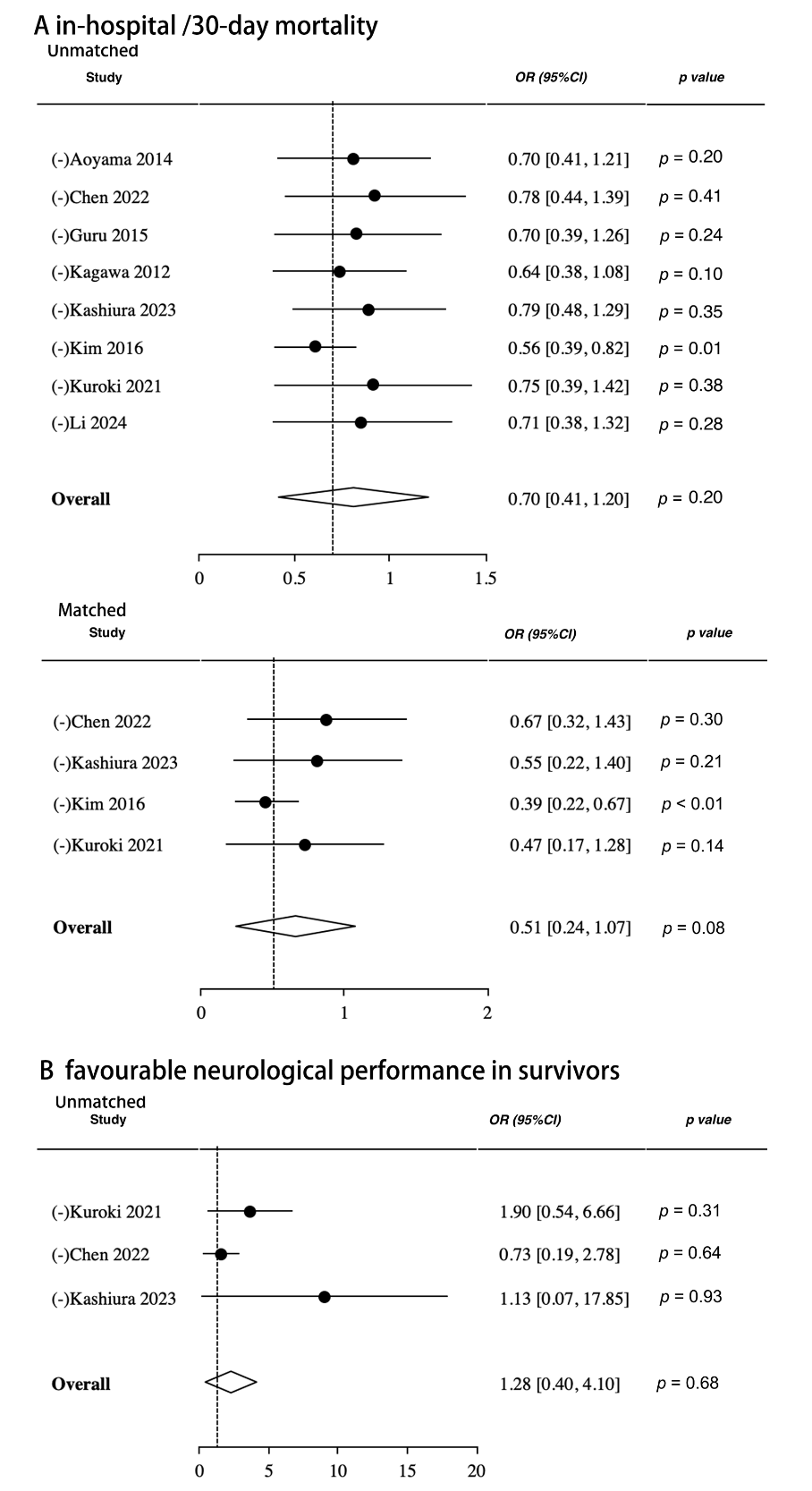


**Supplementary Table 1.** Detailed search strategy.

**PubMed 54**

(((((((((Arrest, Heart[Title/Abstract]) OR (Cardiac Arrest[Title/Abstract])) OR (Arrest, Cardiac[Title/Abstract])) OR (Asystole[Title/Abstract])) OR (Asystoles[Title/Abstract])) OR (Cardiopulmonary Arrest[Title/Abstract])) OR (Arrest, Cardiopulmonary[Title/Abstract])) OR ("Heart Arrest"[Mesh])) AND ((("Extracorporeal Membrane Oxygenation"[Mesh]) OR (((((((((((((((((((((Extracorporeal Membrane Oxygenations[Title/Abstract]) OR (Membrane Oxygenation, Extracorporeal[Title/Abstract])) OR (Oxygenation, Extracorporeal Membrane[Title/Abstract])) OR (ECMO Treatment[Title/Abstract])) OR (ECMO Treatments[Title/Abstract])) OR (Treatment, ECMO[Title/Abstract])) OR (ECLS Treatment[Title/Abstract])) OR (ECLS Treatments[Title/Abstract])) OR (Treatment, ECLS[Title/Abstract])) OR (ECMO Extracorporeal Membrane Oxygenation[Title/Abstract])) OR (Extracorporeal Life Support[Title/Abstract])) OR (Extracorporeal Life Supports[Title/Abstract])) OR (Life Support, Extracorporeal[Title/Abstract])) OR (Venoarterial ECMO[Title/Abstract])) OR (ECMO, Venoarterial[Title/Abstract])) OR (Venoarterial ECMOs[Title/Abstract])) OR (Venoarterial Extracorporeal Membrane Oxygenation[Title/Abstract])) OR (Venovenous ECMO[Title/Abstract])) OR (ECMO, Venovenous[Title/Abstract])) OR (Venovenous ECMOs[Title/Abstract])) OR (Venovenous Extracorporeal Membrane Oxygenation[Title/Abstract])))))) AND ((((((((Intra Aortic Balloon Pumping[Title/Abstract]) OR (Intraaortic Balloon Pumping[Title/Abstract])) OR (Pumping, Intraaortic[Title/Abstract])) OR (Pumping, Intraaortic Balloon[Title/Abstract])) OR (Pumping, Intra-Aortic Balloon[Title/Abstract])) OR (Balloon Pumping, Intra-Aortic[Title/Abstract])) OR (Pumping, Intra Aortic Balloon[Title/Abstract])) OR ("Intra-Aortic Balloon Pumping"[Mesh]))

**Web of Science 133**

TS=(Heart Arrest) OR AB=((Arrest, Heart) OR (Cardiac Arrest) OR (Arrest, Cardiac) OR (Asystole) OR (Asystoles) OR (Cardiopulmonary Arrest) OR (Arrest, Cardiopulmonary))

AND

TS=(Intra-Aortic Balloon Pumping) OR AB=((Intra Aortic Balloon Pumping) OR (Intraaortic Balloon Pumping) OR (Pumping, Intraaortic) OR (Pumping, Intraaortic Balloon) OR (Pumping, Intra-Aortic Balloon) OR (Balloon Pumping, Intra-Aortic) OR (Pumping, Intra Aortic Balloon))

AND

TS=(Extracorporeal Membrane Oxygenation) OR AB=((Extracorporeal Membrane Oxygenations) OR (Membrane Oxygenation, Extracorporeal) OR (Oxygenation, Extracorporeal Membrane) OR (ECMO Treatment) OR (ECMO Treatments) OR (Treatment, ECMO) OR (ECLS Treatment) OR (Extracorporeal Life Support) OR (Extracorporeal Life Supports) OR (Life Support, Extracorporeal) OR (Venoarterial ECMO) OR (ECMO, Venoarterial) OR (Venoarterial ECMOs) OR (Venoarterial Extracorporeal Membrane Oxygenation) OR (Venovenous ECMO) OR (ECMO, Venovenous) OR (Venovenous ECMOs) OR (Venovenous Extracorporeal Membrane Oxygenation))

**Embase 612**

'Extracorporeal Membrane Oxygenation'/exp OR 'Extracorporeal Membrane Oxygenations':ab OR 'Membrane Oxygenation, Extracorporeal':ab OR 'Oxygenation, Extracorporeal Membrane':ab OR 'ECMO Treatment':ab OR 'ECMO Treatments':ab OR 'Treatment, ECMO':ab OR 'ECLS Treatment':ab OR 'Extracorporeal Life Support':ab OR 'Extracorporeal Life Supports':ab OR 'Life Support, Extracorporeal':ab OR 'Venoarterial ECMO':ab OR 'ECMO, Venoarterial':ab OR 'Venoarterial ECMOs':ab OR 'Venoarterial Extracorporeal Membrane Oxygenation':ab OR 'Venovenous ECMO':ab OR 'ECMO, Venovenous':ab OR 'Venovenous ECMOs':ab OR 'Venovenous Extracorporeal Membrane Oxygenation':ab

AND

'Heart Arrest'/exp OR 'Arrest, Heart':ab OR 'Cardiac Arrest':ab OR 'Arrest, Cardiac':ab OR 'Asystole':ab OR 'Asystoles':ab OR 'Cardiopulmonary Arrest':ab OR 'Arrest, Cardiopulmonary':ab

AND

'Intra-Aortic Balloon Pumping'/exp OR 'Intra Aortic Balloon Pumping':ab OR 'Intraaortic Balloon Pumping':ab OR 'Pumping, Intraaortic':ab OR 'Pumping, Intraaortic Balloon':ab OR 'Pumping, Intra-Aortic Balloon':ab OR 'Balloon Pumping, Intra-Aortic':ab OR 'Pumping, Intra Aortic Balloon':ab

**Ovid MEDLINE 26**

Ovid MEDLINE(R) ALL <1946 to February 22, 2024>

1 *Extracorporeal Membrane Oxygenation/ 13263

2 Extracorporeal Membrane Oxygenations.ab,ti. 7

3 Membrane Oxygenation, Extracorporeal.ab,ti. 24

4 Oxygenation, Extracorporeal Membrane.ab,ti. 17

5 ECMO Treatment.ab,ti. 497

6 ECMO Treatments.ab,ti. 15

7 Treatment, ECMO.ab,ti. 11

8 ECLS Treatment.ab,ti. 26

9 Extracorporeal Life Support.ab,ti. 2848

10 Extracorporeal Life Supports.ab,ti. 3

11 Life Support, Extracorporeal.ab,ti. 17

12 Venoarterial ECMO.ab,ti. 441

13 ECMO, Venoarterial.ab,ti. 11

14 Venoarterial ECMOs.ab,ti. 0

15 Venoarterial Extracorporeal Membrane Oxygenation.ab,ti. 1511

16 Venovenous ECMO.ab,ti. 400

17 ECMO, Venovenous.ab,ti. 13

18 Venovenous ECMOs.ab,ti. 0

19 Venovenous Extracorporeal Membrane Oxygenation.ab,ti. 927

20 1 or 2 or 3 or 4 or 5 or 6 or 7 or 8 or 9 or 10 or 11 or 12 or 13 or 14 or 15 or 16 or 17 or 18 or 19 15425

21 Heart Arrest/ 32964

22 Arrest, Heart.ab,ti. 65

23 Cardiac Arrest.ab,ti. 43859

24 Arrest, Cardiac.ab,ti. 148

25 Asystole.ab,ti. 4184

26 Asystoles.ab,ti. 70

27 Cardiopulmonary Arrest.ab,ti. 2807

28 Arrest, Cardiopulmonary.ab,ti. 179

29 21 or 22 or 23 or 24 or 25 or 26 or 27 or 28 62447

30 *Intra-Aortic Balloon Pumping/ 2550

31 Intra Aortic Balloon Pumping.ab,ti. 854

32 Intraaortic Balloon Pumping.ab,ti. 490

33 Pumping, Intraaortic.ab,ti. 1

34 Pumping, Intraaortic Balloon.ab,ti. 1

35 Pumping, Intra-Aortic Balloon.ab,ti. 2

36 Balloon Pumping, Intra-Aortic.ab,ti. 2

37 Pumping, Intra Aortic Balloon.ab,ti. 2

38 30 or 31 or 32 or 33 or 34 or 35 or 36 or 37 3353

39 20 and 29 and 38 26

**Cochrane Library 4**
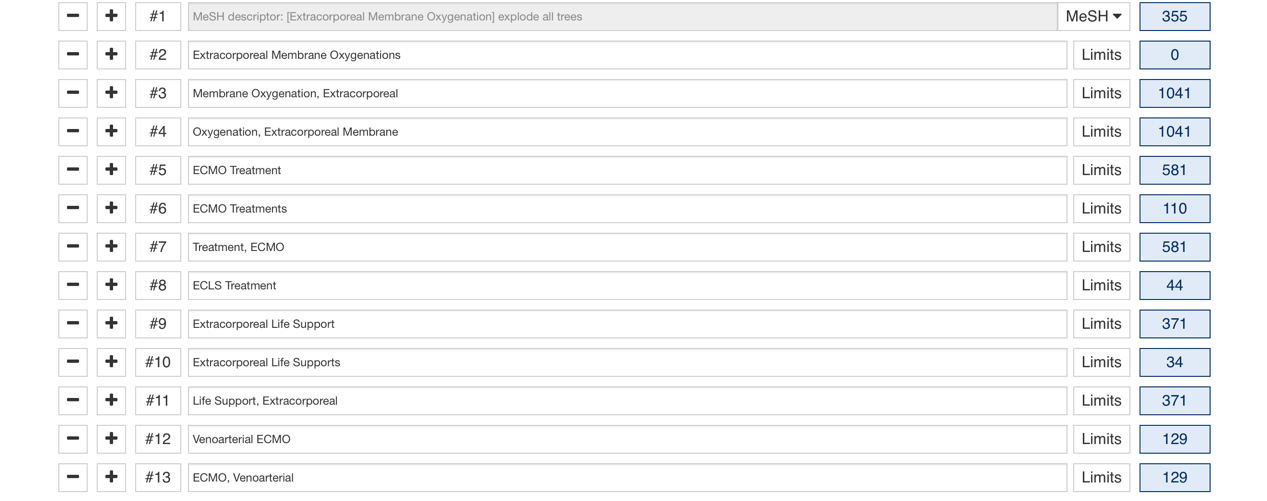


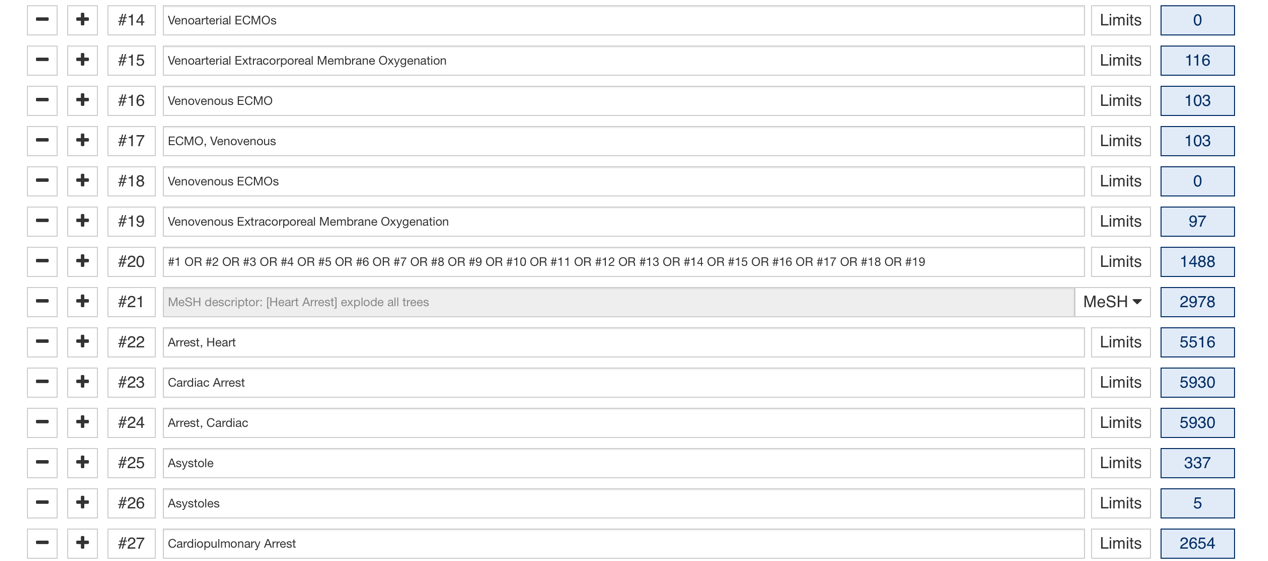

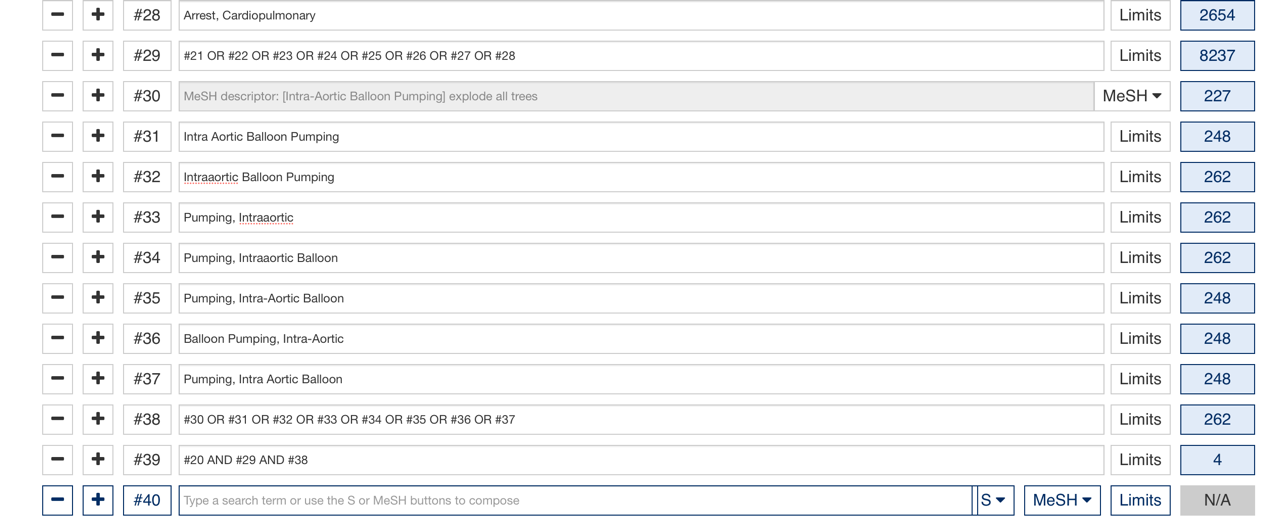


**Supplementary Table 2. Quality assessment**

**Supplementary Table 2.** Quality assessment.

| Study | Representativeness of the exposed cohort | Selection of the non-exposed cohort | Ascertainment of exposure | Outcome not presenting at baseline | Control for age | Control for other confounding factors | Assessment of outcome | Significant follow-up duration | Adequacy of follow-up of cohorts | Total |  |
| --- | --- | --- | --- | --- | --- | --- | --- | --- | --- | --- | --- |
| Kagawa, 2012 | | 1 | 1 | 1 | 1 | 1 | 1 | 1 | 1 | 1 | 9 |
| Aoyama, 2014 | | 1 | 1 | 1 | 1 | 1 | 1 | 1 | 1 | 1 | 9 |
| Guru, 2015 | | 1 | 1 | 1 | 1 | 1 | 1 | 1 | 1 | 1 | 9 |
| Kim, 2016 | | 1 | 1 | 1 | 1 | 1 | 1 | 1 | 1 | 1 | 9 |
| Kuroki, 2021 | | 1 | 1 | 1 | 1 | 1 | 1 | 1 | 1 | 1 | 9 |
| Chen, 2022 | | 1 | 1 | 1 | 1 | 1 | 1 | 1 | 1 | 1 | 9 |
| Kashiura, 2023 | | 1 | 1 | 1 | 1 | 1 | 1 | 1 | 1 | 1 | 9 |
| Xu, 2023 | | 1 | 1 | 1 | 1 | 1 | 1 | 1 | 1 | 1 | 9 |
| Li, 2024 | | 1 | 1 | 1 | 1 | 1 | 1 | 1 | 1 | 1 | 9 |

**Supplementary Table 3.** Patient and treatment characteristics.

|  | | Kagawa  (2012) | Aoyama  (2014) | Guru  (2015) | Kim  (2016) | Kuroki  (2021) | Chen  (2022) | Kashiura  (2023) | Xu  (2023) | Li  (2024) | Total | *P* |
| --- | --- | --- | --- | --- | --- | --- | --- | --- | --- | --- | --- | --- |
| Number of patients | ECPR only | 15 | 35 | 51 | 883 | 173 | 168 | 962 | 50 | 239 | 2576 |  |
|  | ECPR+IABP | 71 | 3 | 50 | 476 | 762 | 31 | 1173 | 6 | 112 | 2684 |  |
| Age, years | ECPR only | N/A | N/A | N/A | 68.5 (12.8) | 64.1 (15.3) | 59.4 (17.5) | 61.0 (17.4) | N/A | N/A | 63.3 (11.3) | < 0.01 |
|  | ECPR+IABP | N/A | N/A | N/A | 68.3 (11.4) | 63.1 (13.6) | 63.7 (11.8) | 62.0 (16.6) | N/A | N/A | 64.3 (8.27) |  |
| Male sex, n (%) | ECPR only | N/A | N/A | N/A | 541 (61.3) | 128 (74.0) | N/A | 719 (74.7) | N/A | N/A | 1388 (68.8) | < 0.01 |
|  | ECPR+IABP | N/A | N/A | N/A | 329 (69.1) | 635 (83.0) | N/A | 1000 (85.3) | N/A | N/A | 1964 (81.5) |  |
| OHCA, n (%) | ECPR only | N/A | N/A | N/A | N/A | 102 (59.0) | 0 (0) | 962 (100) | N/A | N/A | 1064 (81.7) | 0.14 |
|  | ECPR+IABP | N/A | N/A | N/A | N/A | 472 (62.0) | 0 (0) | 1173 (100) | N/A | N/A | 1645 (83.7) |  |
| Document VF/Pulseless VT, n (%) | ECPR only | N/A | N/A | N/A | N/A | 64 (37.0) | 103 (61.3) | 479 (49.8) | N/A | N/A | 646 (49.6) | < 0.01 |
|  | ECPR+IABP | N/A | N/A | N/A | N/A | 298 (39.0) | 10 (32.3) | 791 (67.4) | N/A | N/A | 1099 (55.9) |  |
| PCI, n (%) | ECPR only | 15 (100) | 35 (100) | N/A | N/A | N/A | N/A | 121 (12.6) | N/A | N/A | 171 (16.9) | < 0.01 |
|  | ECPR+IABP | 71 (100) | 3 (100) | N/A | N/A | N/A | N/A | 680 (58.0) | N/A | N/A | 754 (60.5) |  |
| AMI, n (%) | ECPR only | 15 (100) | 35 (100) | N/A | 883 (100) | N/A | N/A | N/A | N/A | N/A | 933 (100) | 0.99 |
|  | ECPR+IABP | 71 (100) | 3 (100) | N/A | 476 (100) | N/A | N/A | N/A | N/A | N/A | 550 (100) |  |
| Prior MI, n (%) | ECPR only | N/A | N/A | N/A | 66 (7.5) | N/A | 25 (14.9) | N/A | N/A | N/A | 91 (8.7) | 0.89 |
|  | ECPR+IABP | N/A | N/A | N/A | 40 (8.4) | N/A | 3 (9.7) | N/A | N/A | N/A | 43 (8.5) |  |
| DM, n (%) | ECPR only | N/A | N/A | N/A | 327 (37.0) | 42 (24.0) | 78 (46.4) | N/A | N/A | N/A | 447 (36.5) | 0.60 |
|  | ECPR+IABP | N/A | N/A | N/A | 187 (39.3) | 249 (33.0) | 14 (45.2) | N/A | N/A | N/A | 450 (35.5) |  |
| HP, n (%) | ECPR only | N/A | N/A | N/A | 504 (57.1) | 67 (39.0) | 78 (46.4) | N/A | N/A | N/A | 649 (53.0) | 0.14 |
|  | ECPR+IABP | N/A | N/A | N/A | 269 (59.5) | 351 (46.0) | 15 (48.4) | N/A | N/A | N/A | 635 (50.0) |  |

ECPR, extracorporeal cardiopulmonary resuscitation; IABP, intra-aortic balloon pump; OHCA, out-of-hospital cardiac arrest; PCI, percutaneous coronary intervention; AMI, acute myocardial infarction; DM, diabetes mellitus; HP, hypertension.
